# Whole-Genome Assemblies for Two Yersinia pestis Strains Isolated in Mongolia

**DOI:** 10.1128/MRA.00920-20

**Published:** 2020-10-08

**Authors:** Muzi Jin, B. Byambajav, Hongyuan Zheng, Yufei Chen, B. Natsagdorj, Chao Yang, Yajun Song, Jing Wang

**Affiliations:** a Huhhot Customs District, Huhhot, China; b National Center for Zoonotic Diseases, Ulaanbaatar, Mongolia; c State Key Laboratory of Pathogen and Biosecurity, Beijing Institute of Microbiology and Epidemiology, Beijing, China; d Chinese Academy of Inspection and Quarantine, Beijing, China; University of Maryland School of Medicine

## Abstract

Here, we report the draft genome sequences of two Yersinia pestis bv. Antiqua strains, belonging to the 3.ANT phylogroup, that were isolated in Mongolia and were circulating in marmot populations.

## ANNOUNCEMENT

Plague is caused by Yersinia pestis, a Gram-negative bacterium that belongs to *Enterobacteriaceae.* It has claimed the lives of millions of people in the three major historical pandemics, and the pathogen has greatly influenced human history on a global scale (1). According to calculations based on a neutral molecular clock, Y. pestis differentiated from its ancestor Yersinia pseudotuberculosis around 6,000 years ago (2). Based on whole-genome-wide variations, Y. pestis strains could be attributed to five major branches (3).

Mongolia is a landlocked nation bordering Russia to the north and China to the south. It is located in central Asia, with an area of about 1.56 million km^2^; the main groups of Y. pestis there are populations of Y. pestis 3.ANT2 phylogroup strains belonging to Y. pestis bv. Antiqua (3). In this study, we isolated two Y. pestis strains from the carcasses of two marmots (Marmota sibirica). Strain 3243 was isolated in 2016 from the brain of a dead marmot in Kusgul Aymag, Mongolia (43073′22.3, 111089′197.1), and strain 3256 was isolated in 2017 from the spleen of a dead marmot in Dzavhan Aymag, Mongolia (43077′61.0, 111084′81.0).

These two isolates were cultured on Hottinger's agar (pH 6.9 to 7.1; prepared at the National Center for Zoonotic Diseases, Mongolia) at 28°C for 2 days and were confirmed with microscopic examinations, a PCR method, and biochemical identification tests. These two isolates were then cultured on Hottinger's agar for 18 h for DNA extraction, and DNA samples were extracted using the Qiagen DNeasy blood and tissue kit.

Sequencing libraries were prepared using the MGIEasy universal DNA library preparation set (BGI, Shenzhen, China), and whole-genome sequencing was performed using an Illumina NovaSeq 6000 system according to the manufacturer’s instructions. For the Illumina sequencing library, the insert size was 350 bp, with a paired-end sequencing length of 150 bp. Finally, we obtained 32,856,824 and 34,512,662 paired-end raw reads for strain 3243 and strain 3256, respectively. We then used Trimmomatic v0.38 (4) to remove low-quality sequencing reads (quality values of >20). After filtering the raw data, we obtained 400 Mb of clean reads for strains 3243 and 3256, with genome coverage values of 86× and 88×, respectively. The paired-end reads were *de novo* assembled by SPAdes v3.12.0 (5), and coding sequences (CDSs) were annotated by Prokka v1.13 (6). All software settings used were the default parameters unless otherwise mentioned. Finally, we obtained 193 and 185 contigs for strains 3243 and 3256, respectively. The genome characteristics of the two strains are recorded in Table 1.

**TABLE 1:** Basic statistics for assemblies and annotations

Strain	Size (bp)	Coverage (×)	No. of contigs	No. of CDSs	G+C content (%)	*N*_50_ (bp)
3243	4,664,220	86	193	4,096	47.53	51,078
3256	4,563,639	88	185	4,014	47.48	53,846

### Data availability.

The whole-genome shotgun projects for strains 3243 and 3256 have been deposited in DDBJ/ENA/GenBank under the accession numbers JAABOF000000000 and JAABOG000000000, respectively. The versions described in this paper are the first versions, JAABOF000000000.1 and JAABOG000000000.1, respectively. The publicly available genome assemblies have been annotated via NCBI PGAP v4.11 (7, 8). The Sequence Read Archive (SRA) data for strains 3243 and 3256 have been deposited in the NCBI SRA under the accession numbers SRR10912556 and SRR10912555, respectively.
